# Supplementary material for: Chemical signals from eggs facilitate cryptic female choice in humans
Source: Proc Biol Sci. 2020 Jun 10;287(1928):20200805. doi: 10.1098/rspb.2020.0805 (PMC7341926; doi:10.1098/rspb.2020.0805)
Supplement: Supplementary methods, tables and figure [file rspb20200805supp1.docx]

**Supplemental Information**

**Chemical signals from eggs facilitates cryptic female choice in humans**

John L. Fitzpatrick^1,2^, Charlotte Willis^3^, Alessandro Devigili^1^, Amy Young^2^, Michael Carroll^4^, Helen R. Hunter^5^ and Daniel R. Brison^3,5^

Affiliations

^1^ Department of Zoology, Stockholm University, Svante Arrhenius väg 18B, 10691, Stockholm, Sweden

^2^ Faculty of Biology, Medicine and Health, University of Manchester, Oxford Road, Manchester, M13 9PT, UK

^3^ Maternal and Fetal Health Research Centre, Faculty of Biology, Medicine and Health, University of Manchester, Manchester Academic Health Sciences Centre

^4^ Department of life Sciences, Faculty of Science and Engineering, Manchester Metropolitan University, Manchester, United Kingdom, M1 5GD

^5^ Department of Reproductive Medicine, Saint Mary's Hospital, Manchester University NHS Foundation Trust, Manchester Academic Health Sciences Centre; Oxford Road, Manchester M13 9WL

**Supplementary Methods**

**Sample collection**

Follicular fluid and sperm samples were obtained from couples receiving assisted reproductive treatment at the Department of Reproductive Medicine at Old St. Mary’s Hospital in Manchester between 2015-2017. All couples undergoing *in vitro* fertilization (IVF) or intracytoplasmic sperm injection (ICSI) treatment were eligible for inclusion in the study, although subsequent data analyses differentiated between IVF and ICSI treatments (described in the *Statistical Analyses* section of the main text). Couples were only included in the study if both partners provided informed written consent. The study was approved under the Human Fertilisation & Embryology Authority (HFEA) research license R0026 for sperm, egg and embryo research (Integrated Research Application System (IRAS) project ID 109131, Research Ethics Committee (REC) reference 12/SC/0649).

Samples were collected using standard WHO clinical practices. Follicular fluid samples were obtained by aspirating follicular fluid from ovarian follicles using transvaginal guided oocyte retrieval from women experiencing standard hormonal stimulation protocols (WHO 2010). The follicular fluid and isolated oocytes for potential use in the participant’s fertility procedure were then screened by clinical embryologists who removed the oocytes from the sample. The remaining follicular fluid was transferred into a collection tube (Vitrolife Collection Tube, Vitrolife, Sweden) and stored at 4°C for up to 48 hours of aspiration before being used in the experiment. Follicular fluid retains its chemoattractant ability for up to 2 weeks of storage (Ralt et al. 1991). For men, semen samples were collected by masturbation within a secured sample procurement room at the IVF clinic in St Mary’s Hospital. Immediately following production, samples were incubated at 37°C to encourage liquefaction. In accordance with the World Health Organization (WHO) guidelines, the samples were analyzed within one hour of ejaculation to determine the sperm count and motility (WHO 2010). Following analysis, the samples were washed in sperm preparation medium (SpermRinse™, Vitrolife, Sweden) and prepared for use in the participant’s fertility procedure in accordance with standard instructions (WHO 2010). Following use, any remaining sperm sample was transferred into a collection tube (Vitrolife Collection Tube) and stored at 4°C for up to 48 hours before being used in the experiment. All equipment and solutions used in experiments were assessed for cytotoxic effects on sperm. The survival index values were satisfactory and met the required standard outlined in the departmental protocol (Critchlow et al. 1989).

Standard clinical procedures attempt to standardize sperm density within ejaculates to 3 x 10^6^ sperm/ml. The standardized sperm density was achieved for all replicates in the simultaneous choice experiment, where the IVF:ICSI patient ratio was skewed towards IVF patients (14 IVF:2 ICSI) who have comparatively higher sperm density (see main text). However, in the non-simultaneous choice experiment we were unable to achieve the desired standardized sperm density for clinical procedures, and instead average sperm density was reduced to 2.29 x 10^6^ sperm/ml (range: 0.60 x 10^6^ - 9.55 x 10^6^ sperm/ml) for these experiments. The reduction in sperm density stemmed from a greater proportion of ICSI patients (30 IVF:14 ICSI) included in non-simultaneous choice experiment, with accompanying reductions in sperm densities. In addition, the non-simultaneous choice experiment required sperm to be added to twice as many experimental replicates as in the simultaneous choice experiment (see Figure 1 in the main text for a visual summary of the design), which required greater dilution of the ejaculate sample to allow a sufficient volume to be available for the experiment. Importantly, while differences in sperm densities among males generated wide variance in sperm density among males and experimental blocks, the density of sperm added was standardized for each male within an experimental block. We accounted for variance in sperm density in statistical models in the non-simultaneous choice experiments when necessary (see Statistical Analyses in the main text).

**Sperm choice assays**

Assays were performed in petri dishes (60 mm, Falcon® IVF Round Dish) containing two 2-μl microcapillary tubes (Blaubrand intraEnd Micropipettes), filled with follicular fluid from the two females and sealed at one end with putty (Critoseal Capillary Tube Sealant). The microcapillary tubes were placed in standardized positions in the petri dish, 15 mm from the side of the petri dish to reduce potential edge effects on sperm movement, and a small amount of putty was used to secure the tubes at the sealed end within the dish. Once the microcapillary tubes were in place, 8 mL of sperm preparation medium (SpermRinse™) was added to the petri dish. Petri dishes containing the microcapillary tubes and sperm preparation medium were then placed in a Sanyo incubator at 37° and 6% CO_2_ and left undisturbed for 15 minutes to generate a chemical gradient of decreasing concentration away from the entrance to the microcapillary tube towards which sperm can orient (see Jeon et al. 2001 for a similar approach). After 15 minutes, 200-μl or 100-μl of washed sperm diluted in sperm preparation medium in the simultaneous and non-simultaneous choice experiments, respectively, was added at a 90° perpendicular angle to the petri dish on a marked position located equidistant between the two capillary tubes at the base of the sealed end of the capillary tubes. The difference in sperm volume between the simultaneous and non-simultaneous choice experiments was to account for the doubling in the number of petri dishes used in the non-simultaneous vs. simultaneous choice experiment. The petri dishes remained in the incubator during the sperm addition to prevent disruption to the chemoattractant gradient. To account for side biases, the position of the microcapillary tubes was reciprocated between experimental replicates.

**Assessing sperm swimming behaviour**

In all treatments used to assess sperm swimming behaviour (partner follicular fluid, non-partner follicular fluid, and a control medium), 2μL of sperm was added to the well of a 12-well MultiTest slide (MP biomedicals) and immediately diluted with 2μL of the appropriate follicular fluid or control solution. Sperm motility was recorded using a Canon EOS 600D digital camera mounted on a Leica DM750 microscope using phase contrast microscopy at 250 X magnification. Videos were captured at 60 frames per second and all videos were recorded within one minute from the addition of either the follicular fluid or control solution. The videos were subsequently analysed using a CEROS sperm tracker (Hamilton-Thorne Research, Beverly, MA, USA). Analyses focused on seven commonly used CASA parameters: the average smooth path sperm velocity (VAP), the observed curvilinear path of the sperm (VCL), the straight line velocity between the start and end point of the sperm path (VSL), the straightness of the sperm path (STR, measured as the ratio of straight line (VSL) to curvilinear (VCL) velocity), sperm path linearity (LIN, measured as the ratio of the smooth path (VAP) to curvilinear (VCL) velocity), the flagella’s beat rate (or beat cross frequency, BCF), and the magnitude of sperm head displacement when swimming (of the amplitude of lateral head displacement, ALH).

**Repeatability in sperm responses**

We assessed the repeatability between replicates of sperm responses to follicular fluids from different females using the GLMM method in the *rptR* package in R (Nakagawa and Schielzeth 2010). Sperm accumulation measures for each female-male combination from experimental replicates were significantly repeatable (simultaneous choice experiment: R = 0.96, 95% CI = 0.92 – 0.98, p < 0.001; non-simultaneous choice experiment: R = 0.97, 95% CI = 0.95 – 0.98, p < 0.001). Therefore, we used the mean values between replicates as the response variable in subsequent analyses.

**Embryo quality scores, pregnancies and live births**

Embryos were cultured to early cleavage stage (day 2/3) or blastocyst stage (day 5). Generally, if participants had less than 2 embryos, transfer was completed on day 2. If participants had less than 4 good quality embryos, transfer was completed on day 3. If participants had 4 or more good quality embryos on day 3, culture was extended to blastocyst stage (day 5). The grading systems for early cleavage stage embryos and blastocysts are different. The UK National External Quality Assessment Service (NEQAS) produced the embryology morphology grading scheme used to assess the early cleavage stage embryos (National Institute for Health and Care Excellence 2013). For the blastocyst stage, the expansion status, intracellular matrix (ICM) and trophectoderm (TE) are graded (Central Manchester University Hospitals NHS Foundation Trust 2016). To calculate the embryo score measure, each couple’s best quality embryo was selected and the embryo grade was multiplied. Data on the blastocyst stage was only available for three of the 16 couples in the simultaneous choice experiment. Therefore, to facilitate comparison among the two experimental treatments we focused our analyses on embryo quality scores derived from the early cleavage stage for both the simultaneous and non-simultaneous choice experiments. In addition, several of the embryos are typically frozen for subsequent embryo transfer, which adds an additional complication to estimates of embryo viability as the freezing process may influence embryo quality. Therefore, our analyses focused only on fresh embryo quality scores, although we obtained qualitatively similar results when we examined results from previously frozen embryos.

We also collected clinical pregnancy outcomes and live birth rates following assisted reproductive treatments. These measures are not synonymous, as clinical pregnancy does not always lead to live births. Clinical pregnancy included cases where patients were scored as biochemically pregnant, miscarried or gave live birth. Live birth rates only included those patients were embryos were carried to term and offspring produced. Once again, we focused solely on the pregnancy and live birth outcomes from fresh embryos to avoid additional complications added by the inclusion of previously frozen and thawed embryos (although here again we found qualitatively similar results when examining pregnancy and live birth success using previously frozen embryos).

**Additional statistical analyses: sperm accumulation and responsiveness models**

The GLMM and LMM models presented in the main text examining if sperm accumulation and responsiveness were influenced by the origin of the follicular fluid in the simultaneous and non-simultaneous experiments were constructed using several random effects (i.e. female ID, male ID, female ID x male ID, block, observation). To explore the impact of this number of random effects on the model results we performed a series of analyses where we reduced the number of random effects present in our models, effectively removing the random effects of female and male identity and their interaction. Specifically, in the simultaneous choice experiment we fitted a GLMM with a logit link function, with sperm accumulation modelled as a binary response variable (contrasting the sperm number in the partner vs. non-partner follicular fluid), with fertility treatment added as a fixed effect and experimental block and observation number (to account for overdispersion) as random effects. For the non-simultaneous experiment, we fitted a LMM with sperm responsiveness (log10 transformation on positivized values) as the response variable, with follicular fluid origin and fertility treatment as fixed effects and experimental block as a random effect.

In all cases, models with reduced random effects generated qualitatively similar findings as those presented in the main text. Specifically, sperm accumulation did not differ between the follicular fluid of the partner or non-partner in the simultaneous choice experiment (GLMM; fixed intercept: Z = -0.35, p = 0.72; fertility treatment effect (IVF vs. ICSI): Z = 0.92, p = 0.36) and sperm responsiveness was not affected by the origin of the follicular fluid (follicular fluid origin: χ^2^ = 0.29, p = 0.59; fertility treatment: χ^2^ = 2.97, p = 0.09).

**Additional statistical analyses: fertility, embryo quality and outlying data points**

In our main analyses we found that IVF fertilization rates (see Fig. S1a) and embryo quality (albeit a statistical trend) were positively related with partner sperm preference in the simultaneous choice experiment. However, these effects appeared to be driven by two outlying data points, showing extreme responses (one positive and one negative) in sperm preference relative to all other couples (Fig. 1a). When these two outliers were removed, the positive relationship previously observed disappeared. The relationship between fertilization rates and sperm preference flipped directions, showing a borderline significant negative relationship that suggested fertilization rates were higher when sperm were less responsive to their partner’s follicular fluid (Z = -1.99, p = 0.05). There was no evidence of a relationship between embryo quality and sperm preference when the two outlier data points were removed (χ^2^ = 1.08, p = 0.30).

**Table S1. Sources of variation in sperm accumulation in the (a) simultaneous choice experiments for IVF patients and in the non-simultaneous choice experiments for (b) IVF patients and (c) ICSI patients**.

| Source of variation | | df | SS | MS | F | p |
| --- | --- | --- | --- | --- | --- | --- |
| *(a) simultaneous choice experiment – IVF patients* | | |  |  |  |  |
|  | female | 6 | 510695.9 | 85116.0 | 0.85 | 0.58 |
|  | male | 6 | 2747549.9 | 457925.0 | 4.59 | **0.04** |
|  | female x male interaction | 6 | 599191.9 | 99865.3 | 30.13 | **<0.001** |
|  | error | 24 | 79550.5 | 3314.6 |  |  |
|  |  |  |  |  |  |  |
| *(b) non-simultaneous* *choice experiment – IVF patients* | | |  |  |  |  |
|  | female | 12 | 7703209.6 | 641934.1 | 10.04 | **<0.001** |
|  | male | 12 | 1865353.1 | 155446.1 | 2.43 | 0.07 |
|  | female x male interaction | 12 | 767319.1 | 63943.3 | 18.39 | **<0.001** |
|  | error | 48 | 166906.5 | 3477.2 |  |  |
|  |  |  |  |  |  |  |
| *(c) non-simultaneous* *choice experiment – ICSI patients* | | |  |  |  |  |
|  | female | 10 | 1655490.2 | 165549.0 | 2.95 | 0.05 |
|  | male | 10 | 546739.3 | 54673.9 | 0.97 | 0.52 |
|  | female x male interaction | 10 | 561032.3 | 56103.2 | 29.30 | **<0.001** |
|  | error | 40 | 76601.0 | 1915.0 |  |  |

Note that thre were too few ICSI patients in the simultaneous choice experiment to allow this subcategory to be investigated. The degrees of freedom (df) and sum of squares (SS) were calculated individually for each experiment block using a series of sequential two-way ANOVAs. The df and SS from all experiment blocks were summed and combined to estimate the mean squares (MS) for each analysis. The df for each block was calculated by multiplying the number of females, number of males, and number of replicate crosses minus one for each block. The dfs from main effects and error estimates were summed across blocks. F values were obtained for male and female effects by dividing their respective MS values by the interaction MS. F values for the interaction term were calculated by dividing the interaction MS value with the error MS. Statistically significant values are in bold. Due to differences in sperm number among males (but not between replicates for each male within an experimental block), we did not interpret male effects in our models, nor did we interpret main effects when significant interactive effects were detected.

**Supplementary Table S2. Principal component analysis of sperm swimming parameters generated from computer assisted sperm analysis in humans.**

|  | Principal component | |
| --- | --- | --- |
| Sperm trait | **PC1** | **PC2** |
| VAP | **-0.44** | -0.18 |
| VSL | **-0.46** | 0.05 |
| VCL | **-0.33** | **-0.54** |
| ALH | **-0.38** | 0.16 |
| BCF | 0.23 | **0.56** |
| STR | **-0.38** | 0.34 |
| LIN | **-0.38** | **0.46** |
|  |  |  |
| Eigenvalue | 2.13 | 1.12 |
| Percentage (%) of variation explained | 65.0 | 17.8 |

Eigenvectors shown in bold are > 70% of the largest eigenvector and contributed significantly to the PC (Mardia et al. 1979).

**Supplementary Table S3. Effects of follicular fluid on sperm swimming behaviour.**

|  | Fixed Effects | χ^2^ | p |
| --- | --- | --- | --- |
| (a) Sperm velocity PC1: follicular fluid vs. control solution | |  |  |
|  | Experimental Medium (follicular fluid vs. control solution) | 10.07 | **<0.01** |
|  | Fertility Treatment | 15.22 | **<0.001** |
|  | Medium * Fertility Treatment | 5.37 | **<0.01** |
|  |  |  |  |
| (b) Sperm velocity PC2: follicular fluid vs. control solution | |  |  |
|  | Experimental Medium (follicular fluid vs. control solution) | 9.49 | **<0.01** |
|  | Fertility Treatment | 0.13 | 0.72 |
|  |  |  |  |
| (c) Sperm velocity PC1: partner vs. non-partner follicular fluid | |  |  |
|  | Follicular fluid origin (partner vs. non-partner) | 0.28 | 0.60 |
|  | Fertility Treatment | 11.24 | **<0.001** |
|  |  |  |  |
| (d) Sperm velocity PC2: partner vs. non-partner follicular fluid | |  |  |
|  | Follicular fluid origin (partner vs. non-partner) | 0.61 | 0.43 |
|  | Fertility Treatment | 0.05 | 0.83 |

Effects estimates are presented from linear mixed effects models on two principal components (PC1 and PC2) describing sperm swimming behaviour (see Table S2). Sperm swimming behaviour was compared (a, b) when swimming in follicular fluid vs. a control solution and (c, d) when swimming in follicular fluid from a partner vs. a non-partner. In all models, fertility treatment (IVF vs. ICSI) and interactions among predictor variables were included as fixed effects and male identity and experimental block were included as random effects. Sperm swam with greater flagella beat rate and in more linear trajectories when swimming in follicular fluid compared to a control solution across all males sampled and swam slower in follicular fluid compared to a control solution for IVF, but not ICSI, patients, suggesting an interesting interaction with male fertility phenotype. These responses in sperm behaviour are likely due to the difference in viscosity between follicular fluid and the control solution. Non-significant interaction terms were removed from final models. Significant effects are in bold text.

**Supplementary Figure S1.**

(a)
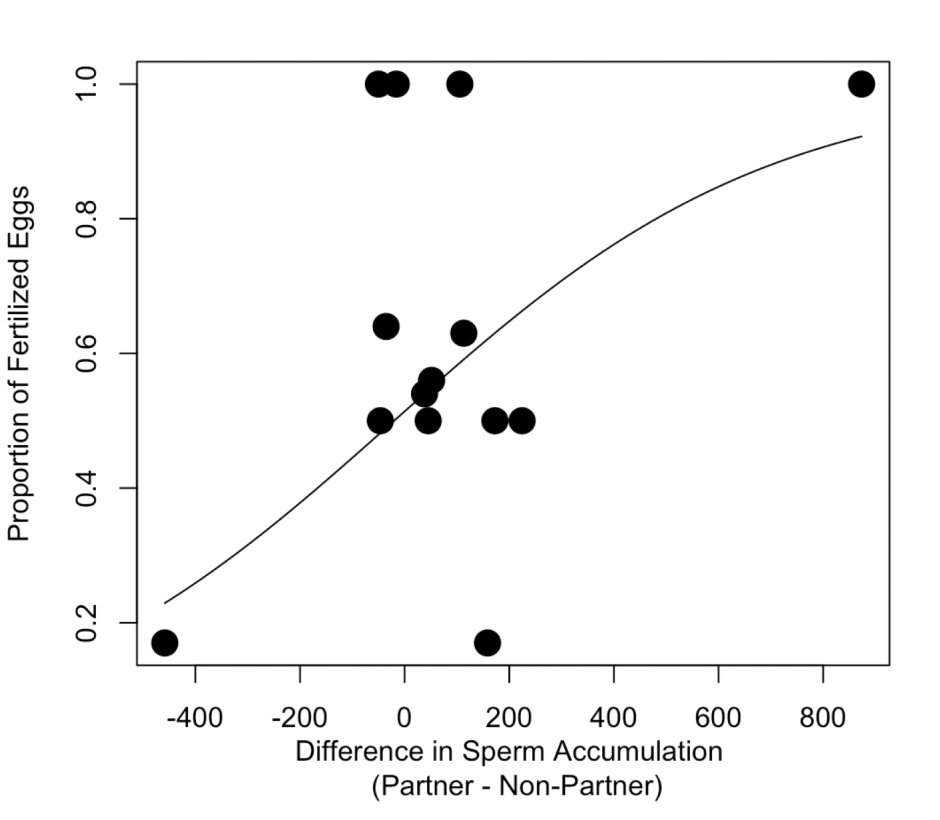


(b)

**Supplementary Figure S1. Fertilization rates and sperm responses to their partner’s follicular fluid.** The relationship between the proportion of eggs fertilized during *in vitro* fertilization (not including ICSI cycles) and (a) the difference in sperm accumulation (partner – non-partner, i.e. partner sperm preference) in the simultaneous choice experiment and (b) the sperm responsiveness (i.e. the difference in sperm accumulation in follicular fluid relative to a control solution between the partner and non-partner) in the non-simultaneous choice experiment.

**Supplementary References**

Central Manchester University Hospitals NHS Foundation Trust. Instruction - Blastocyst Grading (2016).

Critchlow, J. D., Matson, P. L., Newman, M. C., Horne, G., Troup, S. A., & Lieberman, B. A. Quality control in an in-vitro fertilization laboratory: use of human sperm survival studies. *Hum Reprod* **4**, 545-549 (1989).

Mardia, K., Kent, J., & Bibby, J. Multivariate Analysis. Cambridge, Academic Press (1979).

Nakagawa, S. & Schielzeth H. Repeatability for Gaussian and non-Gaussian data: a practical guide for biologists. *Biol Rev* **85**, 935-956 (2010).

National Institute for Health and Care Excellence. UK NEQAS Embryo Morphology Scheme. In *Assessment and Treatment for People with Fertility Problems: NICE Clinical Guideline 156*, pp. Appendix O (2013).

Ralt, D., Goldenberg, M., Fetterolf, P., Thompson, D., Dor, J., Mashiach, S., Barbers, D. L., & Eisenbach, M. Sperm attraction to a follicular factor(s) correlates with human egg fertilizability. *Proc Natl Acad Sci USA* **88**, 2840-2844 (1991).

WHO laboratory manual for the Examination and processing of human semen. World Health Organization (2010).
